# Exploring the Benzazoles Derivatives as Pharmacophores for AChE, BACE1, and as Anti-Aβ Aggregation to Find Multitarget Compounds against Alzheimer’s Disease

**DOI:** 10.3390/molecules29194780

**Published:** 2024-10-09

**Authors:** Martha Cecilia Rosales Hernández, Marycruz Olvera-Valdez, Jazziel Velazquez Toledano, Jessica Elena Mendieta Wejebe, Leticia Guadalupe Fragoso Morales, Alejandro Cruz

**Affiliations:** 1 Laboratorio de Biofísica y Biocatálisis, Sección de Estudios de Posgrado e Investigación, Escuela Superior de Medicina, Instituto Politécnico Nacional, Plan de San Luis y Salvador Díaz Mirón s/n, Casco de Santo Tomás, Miguel Hidalgo, Ciudad de México 11340, Mexico; jazziel_109@hotmail.com (J.V.T.); jemw2009@gmail.com (J.E.M.W.); lety.23fm@gmail.com (L.G.F.M.); 2 Laboratorio de Nanomateriales Sustentables, Sección de Estudios de Posgrado e Investigación, Escuela Superior de Ingeniería Química e Industrias Extractivas, Instituto Politécnico Nacional, Av. Instituto Politécnico Nacional, Lindavista, Gustavo A. Madero, Ciudad de México 07700, Mexico; olvera.valdez.93@gmail.com; 3 Laboratorio de Investigación en Química Orgánica y Supramolecular, Unidad Profesional Interdisciplinaria de Biotecnología, Instituto Politécnico Nacional, Av. Acueducto s/n, Barrio la Laguna Ticomán, Gustavo A. Madero, Ciudad de México 07340, Mexico

**Keywords:** benzazoles, Alzheimer’s disease, beta secretase, beta amyloid, acetylcholinesterase

## Abstract

Despite the great effort that has gone into developing new molecules as multitarget compounds to treat Alzheimer’s disease (AD), none of these have been approved to treat this disease. Therefore, it will be interesting to determine whether benzazoles such as benzimidazole, benzoxazole, and benzothiazole, employed as pharmacophores, could act as multitarget drugs. AD is a multifactorial disease in which several pharmacological targets have been identified—some are involved with amyloid beta (Aβ) production, such as beta secretase (BACE1) and beta amyloid aggregation, while others are involved with the cholinergic system as acetylcholinesterase (AChE) and butirylcholinesterase (BChE) and nicotinic and muscarinic receptors, as well as the hyperphosphorylation of microtubule-associated protein (tau). In this review, we describe the in silico and in vitro evaluation of benzazoles on three important targets in AD: AChE, BACE1, and Aβ. Benzothiazoles and benzimidazoles could be the best benzazoles to act as multitarget drugs for AD because they have been widely evaluated as AChE inhibitors, forming π–π interactions with W286, W86, Y72, and F338, as well as in the AChE gorge and catalytic site. In addition, the sulfur atom from benzothiazol interacts with S286 and the aromatic ring from W84, with these compounds having an IC_50_ value in the μM range. Also, benzimidazoles and benzothiazoles can inhibit Aβ aggregation. However, even though benzazoles have not been widely evaluated on BACE1, benzimidazoles evaluated in vitro showed an IC_50_ value in the nM range. Therefore, important chemical modifications could be considered to improve multitarget benzazoles’ activity, such as substitutions in the aromatic ring with electron withdrawal at position five, or a linker 3 or 4 carbons in length, which would allow for better interaction with targets.

## 1. Introduction

Alzheimer’s disease (AD) is a progressive neurodegenerative disease involving severe damage to cognitive function, which affects activities of daily living. In 2020, it was estimated that more than 55 million people worldwide live with dementia; AD accounted for almost three-quarters of these cases [1]. It has been predicted that, by 2050, the population with dementia and AD will increase to 139 million [2].

The factors involved in the AD pathogenesis have been intensively investigated for several years; however, the biochemical and immunological mechanisms that regulate this disease are still debated. The peptide Aβ has been proposed as a possible initiator that leads to the development of AD, giving rise to the amyloid cascade hypothesis [3]. The amyloidogenic pathway is related to the hydrolysis of the amyloid precursor protein (APP) to release Aβ, which is able to aggregate, forming Aβ oligomers and inducing neurotoxicity [4].

**Figure 1:**
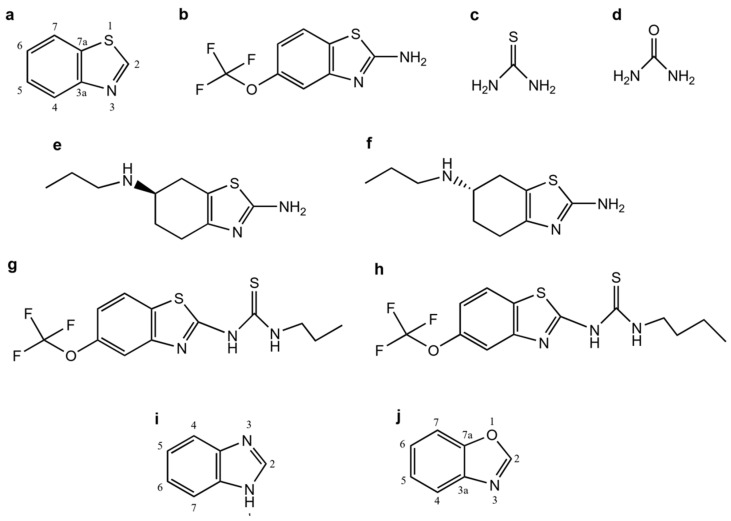
Chemical structures of benzazoles. Benzothiazole (**a**), riluzole (**b**), thiourea (**c**), urea (**d**), dexpramipexole (**e**), and pramipexole (**f**). Derivative compounds from riluzole with tioguanidines compound **3b** (**g**) and compound **3d** (**h**); benzimidazole (**i**) and benzoxazole (**j**).

There are many chemical molecules that have been reported to inhibit Aβ oligomerization, including myricetin, curcumin, resveratrol, etc.; many of these come from natural sources and have principally chemical structures of flavonoids rather than benzazole structures [5]. However, one of the principal molecules employed to identify Aβ is thioflavin (ThT), which has a high affinity for Aβ aggregates [6]. The binding of ThT to diverse fibrils has been studied, suggesting that the rotation of the bond between the carbon from the benzylamine ring and the carbon from position two of the benzothiazole ring allows ThT to produce fluorescence (Figure 1a) due to the interaction of ThT with the β-sheet of the fibril blocked the rotation of this bond by steric hindrance, which increased the ThT fluorescence. Therefore, several in silico studies have been performed, employing peptides with sequences of amino acids that can form β-sheet structures, observing that ThT interacts with aromatic residues as tyrosine and phenylalanine and amino acid residues with a positive charge [7,8].

In addition, a benzothiazole molecule is important in drugs that are employed to treat central nervous system (CNS) diseases such as amyotrophic lateral sclerosis (ALS). For instance, riluzole ((2-amino-6-trifluoromethoxy) benzothiazole) (Figure 1b) has been evaluated due to it possessing a neuroprotective effect and acting on glutamate levels. Furthermore, riluzole has been recently evaluated in AD patients, in whom the glutamate concentration and cognition were determined; however, Aβ was not assayed [9,10]. Furthermore, riluzole was also evaluated in an AD transgenic model, and it was observed that it does not have an effect on Aβ concentration, although it improves cognition in animals [11]. Anzini et al. synthesized riluzole derivatives with guanidine and thiourea (Figure 1c,d) to find compounds with better neuroprotective effects and antioxidant properties. They found that thiourea (Figure 1c) derivatives have a neuroprotective effect and antioxidant activity, with **3b** and **3d** being the best derivatives (Figure 1e,f) [12].

In addition, other molecules that contain a benzothiazole group are used for treating Parkinson’s disease, such as pramipexole (Figure 1g), which is effective as a monotherapy in the early stage of the disease and as adjuvant therapy in the advanced stage [13]. In addition, pramipexole has been evaluated in individuals with mild to moderate AD [14]. Also, preliminary data suggest that dexpramipexole (Figure 1h), which is an aminobenzothiazole derivative, may affect eosinophil maturation in the bone marrow, although its exact mechanism of action is unknown [15,16].

Therefore, the search for a possible molecule to delay or stop AD progression is of great importance. Thus, interest has increased in the design of different scaffolds that could have effects on AD targets, such as beta secretase (BACE1), AChE, and anti-Aβ aggregation, as well as on the oxidative stress produced during AD. Some of them are not only based on benzothiazole; other benzazoles, such as benzimidazoles and benzoxazoles, have also been employed (Figure 1i,j). In this review, we present the molecules with benzazole groups that have been evaluated as inhibitors of BACE1, AChE, and BChE, as well as anti-Aβ aggregation, to test whether they could be useful as multitarget drugs for AD treatment.

The importance of this review lies in the possibility that benzazoles, such as benzothiazole and benzimidazole, act as multitarget compounds to treat AD, a multifactorial disease that does not yet have an effective treatment. Several therapeutic targets have been identified, and this review highlights that benzazoles have the potential to inhibit multiple key targets, such as AChE, BACE1, and Aβ aggregation. A novel finding that has been documented in the literature is that benzimidazoles have demonstrated a high affinity for BACE1, with IC_50_ values in the nM range, which could open a new avenue to develop more effective and specific drugs to treat this disease. This innovative aspect should be underlined, as the evaluation of these molecules as BACE1 inhibitors represents a promising alternative in the search for multitarget treatments for AD.

## 2. Methodology

The information of this review was systematically analyzed to present molecules with benzazole groups that have been evaluated as BACE1, AChE, and BChE inhibitors, as well as anti-Aβ aggregation, to analyze whether they could be useful as multitarget drugs for AD treatment. The search was done considering the words Alzheimer + benzazoles; benzothiazole + Acetylcholinesterase inhibition; Benzothiazole + beta amyloid aggregation; benzothiazole + beta secretase; benzimizole + Acetylcholinesterase inhibition; benzimidazole + beta amyloid aggregation; benzimidazole + beta secretase; benzoxazoles + Acetylcholinesterase inhibition; benzoxazole + beta amyloid aggregation; benzoxazole + beta secretase; benzazoles as multitarget drugs. The search was conducted using Pubmed and Google Scholar. All articles about benzazole and some of the mentioned targets were included. Most articles are from 10 years ago, but there are also some articles about benzazole on BACE1 since 2010, although there is limited information.

## 3. Multitarget Therapy for Alzheimer’s Disease and the Principal Targets

Multitarget therapy involves the development and evaluation of compounds that can act on several targets involved in the same disease. Over the past decade, important efforts have gone into developing multitarget compounds for AD, many of them focused on combining pharmacophores already used for the disease, such as the combination of rivastigmine and rasagline to yield ladostigil [(3R)-3-(prop-2-ynylamino)-2,3-dihydro-1*H*-inden-5-yl] *N*-ethyl-*N*-methylcarbamate, which was created as an AChE and monoamine oxidase (MAO-A and -B) inhibitor also having an effect on oxidative stress and reaching clinical trial phase II [17,18]. However, many drugs that have reached clinical phase III for AD are focused on the Aβ hypothesis but are not proposed as multitarget drugs and do not contain a benzazole scaffold. Currently, there are many peptides proposed as monoclonal antibodies also that act on the Aβ peptide [19,20].

Other molecules in clinical phase III trials that are not based on anti-Aβ therapy in AD but have similar chemical groups to benzazole [20] are OPC-34712 (brexpiprazole), EVP-6124, and troriluzole, which has a benzothiazole molecule [21,22,23]. Recently, an interesting review was published based on compounds that could be multitarget drugs for AD. Many of these compounds act as AChE, butirylcholinesterase (BChE), MAOs, BACE1 inhibitors, and anti-Aβ, along with having antiradical activity. However, although several compounds have been mentioned, only one of them is a benzazole derivative: 2-[4-(4-subtituted-piperazin-1-yl)phenyl]benzimidazole [24].

There are important pathways that have been proposed to explain the AD pathology as an amyloid cascade, the hyperphosphorylation of microtubule-associated protein (tau), and the cholinergic hypothesis, as well as other mechanisms including mitochondrial damage. Of these pathways, the most important for the treatment of AD has been the amyloid cascade, the hyperphosphorylation of tau, and the cholinergic hypothesis; thus, several drugs have been designed to inhibit proteins involved in these pathways.

The cholinergic system is one of the most important physiopathological pathways studied in AD. The limbic and neocortical cholinergic innervation incurred during AD is the central point of the cholinergic hypothesis. The death of cholinergic neurons is related to neurofibrillary degeneration, producing presynaptic cholinergic denervation and causing impairment in cognition [25]. In addition, the cholinergic system has been related to the anti-inflammatory pathway: when the alpha-7 nicotinic receptors (nAChRs) in microglia are activated by acetylcholine (ACh), they avoid the release of proinflammatory cytokines tumor necrosis factor (TNF-alpha), interleukin 1β (IL-1β), and interleukin 6 (IL-6)) and consequently oxidative stress production [26]. Therefore, regulation in the cholinergic system not only occurs via the inhibition of AChE, but also via the regulation of the cholinergic receptors [27]. However, current AD treatment is centered on the inhibition of AChE, which is a cholinergic enzyme whose principal function is to hydrolyze ACh to finish cholinergic neurotransmission. Therefore, in AD, it is necessary to inhibit AChE to increase the amount of ACh in the CNS [28,29]. Another important cholinesterase enzyme, BChE, is a serine hydrolase such as AChE and is also related to cholinergic function, as this enzyme is important in astrocytes and microglia. BChE is also associated with Aβ plaques in the brain cortex in AD [30]. Several molecules that inhibit AChE also inhibit BChE.

MAO-B is an enzyme involved in dopamine metabolism and also in free radical production [31]. Astroglia cells of AD patients express high levels of MAO-B but not MAO-A [32]. The MAO-B increase is principally observed in reactive astrocytes. In addition, a correlation has been observed between MAO-B expression and Aβ production [33]. Although MAO-A is not increased, it is also important in AD because this enzyme catalyzes the deamination reaction of amines, leading to the production of hydrogen peroxide (H_2_O_2_), which is a reactive oxygen species (ROS) that contributes to oxidative stress and cellular death [34].

Another important target in AD is beta-site amyloid precursor protein cleaving enzyme 1 (BACE1), which is an aspartyl protease transmembrane; this enzyme is responsible for producing Aβ, which is an important biomarker responsible for neuron death. Aβ is a fragment of the amyloid precursor protein (APP) that contains between 39 and 42 amino acid residues; that with 42 amino acids (Aβ_1–42_) is more neurotoxic than the others. During AD, there is an alteration in Aβ formation, contributing to the neurotoxicity [35]. Therefore, BACE1 is an important target because its inhibition may prevent Aβ production [36].

In vivo and in vitro studies have associated high levels of Aβ, mainly in oligomeric form, with alterations in glutamatergic synaptic transmission and loss of synapses [37]. Shankar et al., in 2007, demonstrated that physiological concentrations of naturally secreted Aβ dimers and trimers induce a progressive loss of synapses in the hippocampus by modulating an NMDA-type glutamate receptor-dependent signaling pathway [38]. Similarly, Li et al., in 2009, reported that soluble oligomers of Aβ facilitate long-term synaptic depression of the hippocampus by disrupting neuronal glutamate uptake [39].

In the amyloidogenic pathway, BACE1 is an important target, and different inhibitors have been evaluated. Some of these have different chemical groups, such as 2-aminopyridine, acyl guanidine, amino/iminohydatoin, aminoimidazole, aminothiazoline, aminopyrrolidine, and aminoquinoline [40]. It is important to mention that the chemical group guanidine has been important during the design of BACE1 inhibitors because this group allows interactions in the BACE1 active site, forming hydrogen interactions with the aspartic acid of the catalytic site that stabilize the protein in an open conformation [41].

In addition, phosphorylation is a relevant process in AD due to tau’s involvement in microtubule stability. Hyperphosphorylated tau avoids interacting with the microtubules and produces neurofibrillary tangles (NTFs), contributing to the AD pathogenesis. There are several kinases involved in tau phosphorylation, among them glycogen synthase kinase 3-beta (GSK-3β), which can phosphorylate serine and threonine amino acid residues in tau and other proteins [42]. Therefore, tau participates in tubulin assembly, which depends on tau phosphorylation. In AD, this protein is hyperphosphorylated and avoids the stabilization of microtubules, leading to a loss of neuron integrity and allowing NTFs to form [43].

Therefore, in this review, we will focus on the evaluation of benzothiazole, benzimidazole, and benxozazol derivatives for the inhibition of AChE and BACE1, and as anti-Aβ aggregation agents, considering that these compounds could act in multitarget ways. In silico and in vitro studies are mentioned through each series of compounds as has been reported for other hybrid molecules of thiadiazole-based benzothioate and benzenesulfonothioate acting on beta-glucorinadse enzyme whose activity has been determinate also in AD [44,45].

## 4. Benzazole Compounds as AChE Inhibitors

### 4.1. Benzoxazole Derivatives as AChE Inhibitors

**Figure 2:**
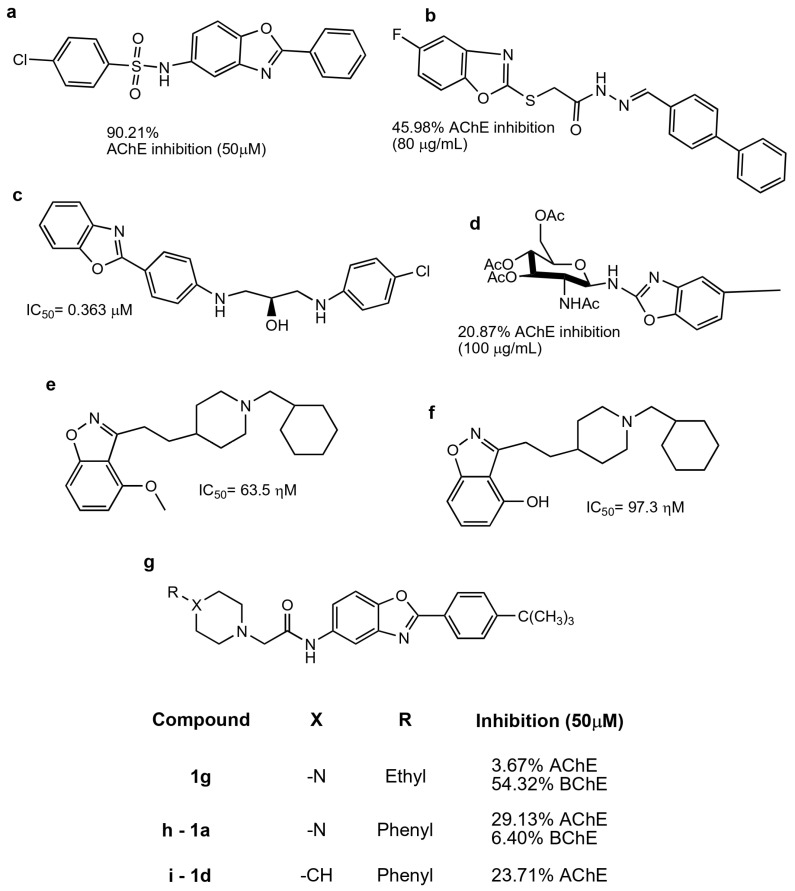
Chemical structures of benzoxazole derivatives used as AChE and BChE inhibitors. Compounds **11** (**a**) [46], **3g** (**b**) [47], **34** (**c**) [48], **5f** (**d**) [49], **32a** (**e**) [50], **33** (**f**) [50], **1g** (**g**) [51], **1a** (**h**) [51], and **1d** (**i**) [51].

Benzoxazole derivatives have been synthesized and evaluated biologically as inhibitors of AChE and BChE. In 2016, Temiz et al. evaluated 13 2,5-substituted benzoxazole derivatives at 50 µM; their compound **11** (Figure 2a) exhibited 90.21% and 68.58% AChE and BChE inhibition, respectively [46]. Its inhibitory activity was measured by some modifications of Ellman’s spectrophotometric method, using galantamine as a reference drug that has an inhibitory effect of 98.15% for AChE and 68.67% for BChE. The affinity for these compounds in the active site of recombinant human AChE was predicted by a molecular docking study. Also, the formation of hydrogen bonds between compound **11** and Y124 was demonstrated. Then, Temiz et al. concluded that the best compound was with a p-substituted sulfonylamido group at the fifth position of 2-(*p*-substitued phenyl) benzoxazole ring.

Altintop et al. synthesized seven hydrazone derivatives (**3a**–**g**) [47]. The structures of compounds were characterized using IR, ^1^H NMR, ^13^C NMR, and mass spectrometry and confirmed via elemental analysis. An MTT (2-(2,5-diphenyl-1*H*-tetrazol-1-ium-3-yl)-4,5-dimethyl-1,3-thiazole; dibromide) assay showed that compound **3g** is not cytotoxic for the NIH-3T3 cell line. Meanwhile, Ellman’s spectrophotometric method was used to determine the inhibitory effects on AChE, using galantamine as a positive control. The results showed that compound **3g** (Figure 2b) is the most effective AChE inhibitor (45.98 ± 3.13%) at 80 µg/mL. Additionally, they used Molinspiration software to determine the molecular properties, considering Lipinski’s rule. Compound **3g** violated one rule of five because it has a logP = 5.46, and this compound may have bioavailability problems.

Several reports indicated that benzoxazole derivatives are effective AChE inhibitors and have potential antioxidant activity. In addition, they enhance learning and memory [48]. Srivastava et al., in 2019, considering Gaussian-based QSAR and virtual screening methods, designed and synthesized phenyl benzoxazole derivatives capable of inhibiting AChE and having antioxidant activity. They designed 292 compounds based on a contour plot and Craig plot, analyzed them, and selected 24 compounds from pose screening and a MM-GBSA method to estimate the free energy between the ligands and AChE. Then, they predicted the binding affinity between the ligand and protein and realized a molecular docking study applying a hybrid sequential combination (Q-SAR and docking). Compounds were synthesized and characterized to determine their antioxidant activity and their half-maximal inhibitory concentration (IC_50_) values using Ellman’s method. Compound **34** (Figure 2c) is better than the others in almost all stages; for example, in silico molecular docking exhibited hydrophobic interactions with I287, F331, W84, F288, Y334, and Y321; electrostatic interactions occurred between the phenyl group and W84 at the catalytic anionic site (CAS); and π–π interactions occurred, while the benzoxazole ring reached the peripheric anionic site (PAS), forming π–π interactions at the active site of AChE. Compound **34** has an IC_50_ of 0.363 ± 0.017 μM in comparison with donepezil’s of 0.04 ± 0.01 μM; both are compounds that are more selective to AChE inhibition than to BChE inhibition with a selective index of 6.3 ± 0.4 and 381 ± 6.33, respectively.

The 1,1-diphenyl-2-picrylhydrazyl (DPPH) method was used to determine the antioxidant activity of all compounds. Compound **34** has antioxidant activity (49.6%) and is closer to ascorbic acid (56.7%). Also, compound **34** was tested in vivo. A Y-maze test showed that this compound has the same effect at 5 mg/kg compared to donepezil (1 mg/mg). Moreover, the ex vivo study revealed a high ACh level in groups treated with donepezil and compound **34**, in contrast with the scopolamine-treated group.

In order to discover new AChE and BChE inhibitors, Wu et al. designed and synthesized some glycosyl benzoxazole derivatives, considering that glucosamine is an anticholinesterase molecule and has antioxidant properties. Ellman’s method was used to evaluate their inhibition activities in vitro [49]. As a result, compound **5f** (Figure 2d) has the best AChE inhibition activity (20.87 ± 0.05%), and compound **5d** is better than the others as a BChE inhibitor (26.91 ± 0.01%) at a concentration of 100 µg/mL.

Benzisoxazole derivatives are potential AChE inhibitors. For example, Lalut et al. used them as an AChE inhibitor and serotonergic 5-HT_4_R agonist [50]. They synthesized compounds to evaluate in vitro and performed docking studies on hAChE and 5-HT_4_R. To evaluate the inhibitory capacity of compounds **11**–**16**, **21**–**23**, **32a**–**e**, and **33** on AChE, they used the spectrometric method of Ellman at a 10^−6^ M concentration. Compounds **32a** and **33** (Figure 2e and Figure 2f, respectively) showed the best AChE-inhibitory activity, with IC_50_ values of 63.5 nM and 97.3 nM, respectively, and K_i_ values of 59 nM and 37 nM for the 5-HT_4_R. Based on these results, they selected both compounds to determine their pharmacological profile by quantification of cAMP production in COS-7 cells. The IC_50_ values obtained were 97.2 ± 17.2 nM and 883.0 ± 597.8 nM, respectively. Finally, the authors concluded that compounds with an ether link have an affinity for 5-HT_4_R, while compounds with a two-carbon methylene link are better AChE inhibitors (Figure 2e,f).

In a study by Celik et al., eight benzoxazole derivatives were evaluated as AChE, BChE, and tyrosinase inhibitors [51]. The AChE and BChE activity were measured using Ellman’s spectrophotometric method, using galantamine as a control drug. Maestro 11.5 and AutoDock Vina 1.1.2. were used for molecular docking studies, using the X-ray crystal structure of human bBChE from PDB (PDB ID: 4BDS). Also, the ADMET profiles and their physicochemical properties were predicted. The compounds were evaluated at 50 μM for AChE and BChE inhibition, and the results showed that **1g** has better BChE inhibition (54.32%) than AChE (3.67%) (Figure 2g). Compound **1a** showed inhibitory activity for AChE (29.13%) and BChE (6.40%), but **1d** only showed inhibitory activity on AChE (23.71%) (Figure 2g). Molecular docking study results from AutoDock Vina showed binding energy values of −9.9, −11.2, and −9.9 kcal/mol for compounds **1a**, **1d**, and **1g**, respectively, higher than the energies obtained with XP GScore (−4.688, −2.999, and −6.385 kcal/mol for **1a**, **1d**, and **1g**, respectively). Finally, not all the compounds conformed to the LogP of Lipinski’s rules, but **1g** showed a value of 5.17.

In conclusion, few benzoxazole molecules are proven AChE inhibitors, and the benzisoxazole derivatives could be better AChE inhibitors. However, the tertiary amine in the piperidine ring could be important for improving the IC_50_ for both molecules, and the presence of a hydrocarbonate chain as a linker may also be essential.

### 4.2. Benzothiazole Derivatives as AChE Inhibitors

**Figure 3:**
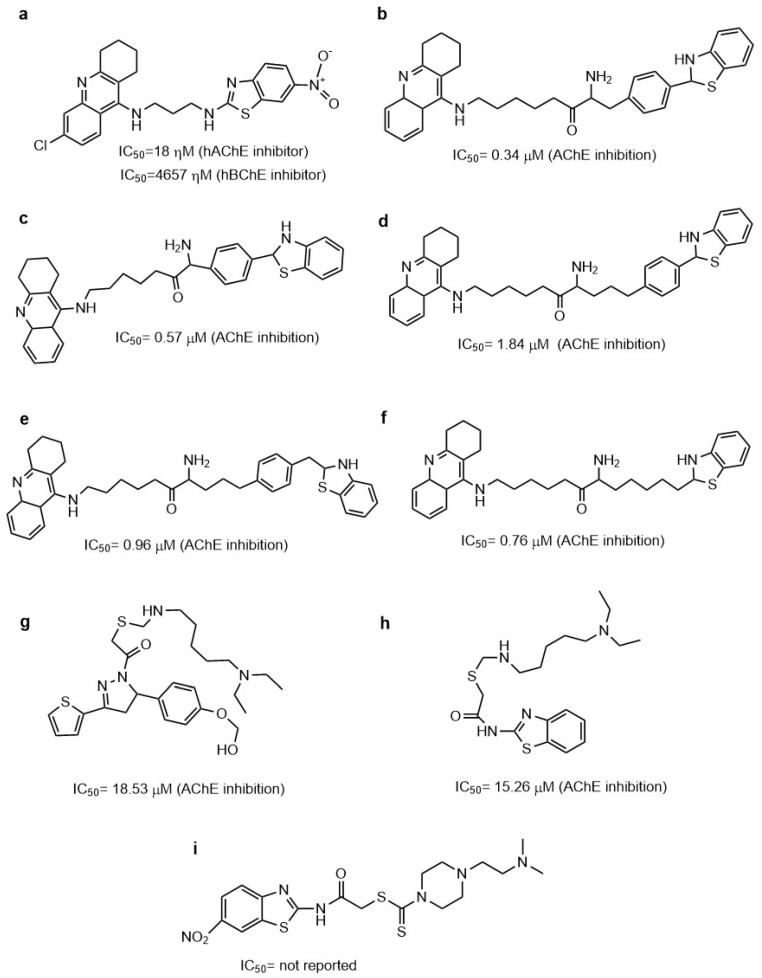
Chemical structures of benzothiazole compounds used as AChE inhibitors. Compounds **10w** (**a**) [53], **7a** (**b**), **7b** (**c**), **7c** (**d**), **7d** (**e**), **7e** (**f**) [54], **A5** (**g**), **A13** (**h**) [55], and **BPCT** (**i**) [56].

The first anticholinesterase molecule approved for the treatment of mild to moderate Alzheimer’s disease was tacrine. Although this drug was a good AChE inhibitor, it was withdrawn from the market in 2013 due to the hepatotoxic effect associated with serum aminotransferase elevation [52]. Studies have employed the tacrine molecule to obtain new tacrine–benzothiazole hybrids in which the length and functional group in the linker between these molecules was modified to be an inhibitor of AChE and Aβ, and to act on mitochondrial systems, showing that compound **10w** (Figure 3a) was the best [53]. In previous research, it was reported that **7a**–**e** compounds (Figure 3b–f) act as AChE and Aβ_1–42_ aggregation inhibitors and protect cells from the death induced by Aβ_1–42_. However, the best activity was not found for any one compound. In addition, all the compounds (**7a**–**e**) showed low antioxidant activity (1 mM scavenger DPPH radical) [54].

Furthermore, the benzothiazole group has been bound with thiophene-2-pyrazoline to obtain new derivatives targeting AChE and (MAO-A)/(MAO-B), showing that compounds **A5** and **A13** (Figure 3g,h) have inhibitory activity against AChE [55]. Here, it is possible to observe that a large hydrocarbon chain did not favor the interaction with AChE due to compounds **A5** and **A13** having higher IC_50_ values than compounds **10w** and **7a**–**e**, which have aromatic rings at the end of each site of the molecules.

The compound 2-[(6-nitro-2-benzothiazolyl)amino]-2-oxoethyl4-[2-(N,N-dimethylamino)ethyl] piperazine-1 carbodithioate (**BPCT**, Figure 3i) was evaluated as an AChE inhibitor in an AD model induced with streptozotocin (STZ). **BPCT** was able to prevent the damage produced by STZ on memory and learning, showing similar results to the animals treated with 3 mg/kg of donepezil (positive control). Also, **BCPT** was evaluated in silico on AChE and was observed to interact with amino acids from the active site, such as Y72, W86, S203, W286, and F338. The piperazine group from **BPCT** forms π–π interactions with the indole ring system of W286. Also, hydrogen bonds form between the oxygen atom of the nitro group and the amino group of S203, and between the oxygen atom of the nitro group and the amino group of W86. The phenyl group from the benzothiazole interacts with F338 by π–π interactions. The ethyl group between the piperazine moiety and terminal nitrogen atom intensifies the binding within the active site by setting up Van der Waals interactions [56,57].

In addition, new compounds from benzoxazolone and benzothiazolone groups with multifunctional activity for AD have been designed and evaluated. From the **8a**–**n**, **9a**–**n**, **10a**–**c**, **11a**–**c**, **14a**–**c**, and **21a**–**b** compounds, the **14b** and **11c** compounds were selected because these presented the highest percent of AChE and BChE inhibition at 100 μM. Afterward, these were evaluated as anti-inflammatory molecules, as well as for their anti-Aβ aggregation power. Results showed that molecules with a benzothiazolone core are better than benzoxazolone; therefore, compounds **14b** and **11c** had better multifunctional activity. Through in silico studies, it was demonstrated that compound **14b** (Table 1) has a binding affinity value of −9.8 kcal/mol for AChE, with the aromatic ring of the benzothiazolone group principally interacting with W279, the sulfur with S286, and the aromatic ring from the chain R with W84 [58].

Table 1 shows other molecules with a benzothiazole ring that have been evaluated as AChE inhibitors, indicating the binding mode, the AChE crystal used, and the IC_50_ or Ki obtained by in vitro studies.

**Table 1:** Benzothiazole derivatives evaluated using molecular docking on AChE.

Compound	In Silico Studies	In Vitro Studies	Refs.
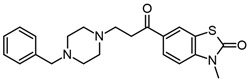 Compound **14b**	Molecular docking on AChE (PDB 1EVE). Amino acids of interactions W84, E199, F330, F331, F290, Y334, W279, and 286. Binding free energy: −9.8 kcal/mol.	IC_50_ 0.34 μM Ki de 0.40 μM Inhibition of βA_42_ 57.5%	[58]
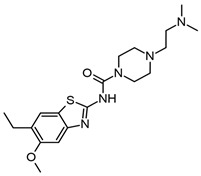 Compound **19**	Molecular docking on AChE (PDB: 4EY7). Compounds **19** and **20** have interactions in the active site. Amino acids of interactions W86, Y124, S203, W286, H287, L289, and Y371.	IC_50_ 0.0462 μM Ki of 0.11 μM	[59]
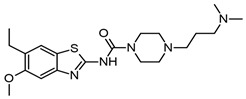 Compound **20**		IC_50_ 0.0576 μM Ki 0.25 μM	[59]
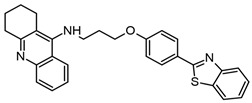 Compound **44b**	Molecular docking (PDB 2CMF) Amino acids of interaction W84, F330, Y70, D72, W271, Y334, F331, and W279.	IC_50_ 0.017 μM Inhibition of Aβ aggregation of 51.8%	[60]

### 4.3. Benzimidazole Derivatives as AChE Inhibitors

**Figure 4:**
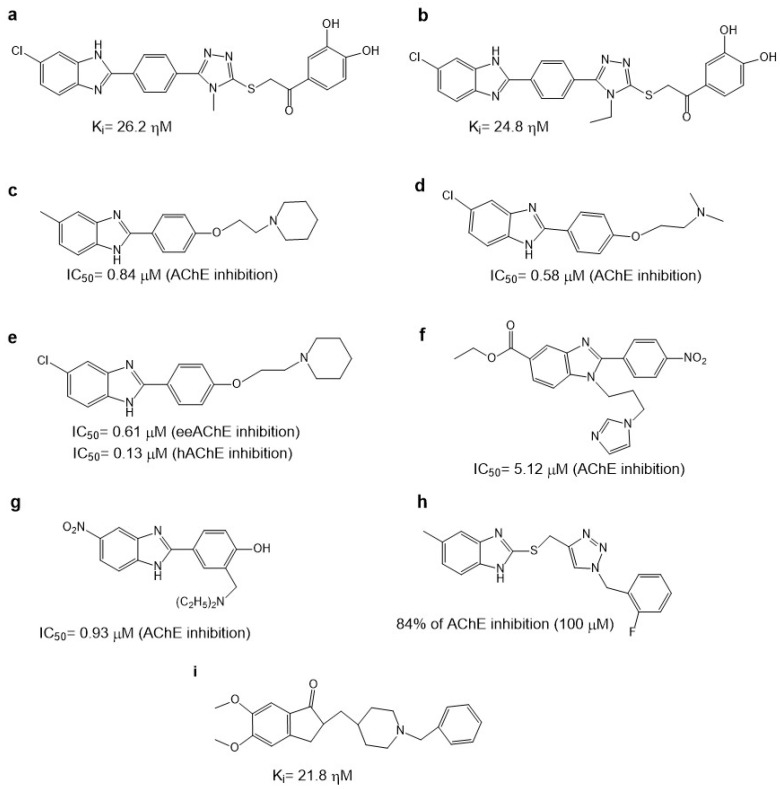
Chemical structures of compounds **3d** (**a**), **3h** (**b**) [61], **2e** (**c**), **3c** (**d**), **3e** (**e**) [62], **5IIc** (**f**) [63], **4b** (**g**) [64], **7e** (**h**), and of donepezil (**i**) [65].

The benzimidazoles are an important group of benzazoles with two atoms of nitrogen (N), one in the 1-position and the other in the 3-position (Figure 1i). The benzimidazoles are good AChE inhibitors. Cevik et al. reported 16 new benzimidazole derivatives such as anti-cholinesterase. Compounds **3d** and **3h** (Figure 4a,b), which have in the triazole ring a 3,4-dihydroxy phenyl ring and in benzimidazole have a 5-chloro substitution, were found to be potent inhibitors of AChE. Their Ki values were 26.2 nM and 24.8 nM for **3d** and **3h**, respectively. These values are comparable to the value obtained for donepezil (21.8 ± 0.9 nM), used as a control. Both compounds were evaluated as AChE inhibitors, employing a modified Ellman’s method [61]. The mode of **3d** and **3h**’s binding to the active site of AChE (*Homo sapiens*: hAChE PDB ID:4EY7) was like dopenezil’s binding: the 5,6-dimethoxyindanone moiety binds to the PAS, interacting with W286 and F295, and the benzylpiperidine group binds to the CAS, interacting with W86.

Compounds **3d** and **3h** had interesting binding modes with the catalytic sites of AChE via the 5(6)-chlorobenzimidazole ring with the phenyl of W286 through π–π interactions. The nitrogen atom of the trizole forms a hydrogen bond with the hydroxyl of Y124. The 3,4-dihydroxyphenyl ring forms π–π interactions with the indole phenyl of W86. These dihydroxy substituents are important for polar interactions. The hydroxyl groups in the 3- and 4-positions form hydrogen bonds with G120, Y133, and E202. However, the differences in binding mode between **3d** and **3h** are not due to the differences in their substituents; **3d** has a methyl and **3h** has an ethyl, so the differences in binding could be due to small differences in the conformational direction. Also, **3h** could form an additional hydrogen bond. In addition, between the compounds evaluated, the substitution of a chlorine atom for a fluorine atom on the benzimidazole ring allows for better inhibition of AChE [61].

Therefore, when the binding of new compounds is analyzed, it is important to consider that the AChE crystal could be from different species, such as Torpedo Californica AChE (TcAChE) and human AChE (hAChE). Alpan et al. published a series of 2-phenyl substituted-1H-benzimidazole derivatives because these are ring isosteres of indanone of donepezil, which is one of the most important AChE inhibitors [62]. They reported that the recognition and effect of these derivatives are different in electric eel acetylcholinesterase (eeAChE) and hAChE. From the 20 derivatives, they identified compounds **2e**, **3c**, and **3e** (Figure 4c–e) as good eeAChE inhibitors with IC_50_ values of 0.84, 0.58, and 0.61 μM, respectively. However, the authors compared these compounds with tacrine (IC_50_ = 0.075 μM) and not with donepezil. In addition, they observed that when a N atom was added in the chain of the p-hydroxyphenol derivatives, the inhibitory activity was improved, except in compounds with morpholine. However, a substitution in the 5-position of the benzimidazole ring with electron-withdrawing or electron-donating does not influence the eeAChE inhibitory activity. However, it was shown that these substitutions produce better inhibitory activity in hAChE due to compound **3e** substituting a chlorine atom in the 5-position of the benzimidazole ring and piperidine on the side chain, giving an IC_50_ of 0.13 μM on hAChe. Docking studies of **3e** showed that the benzimidazole moiety interacts with W279 and Y334 by π–π interactions in the peripheral pocket (PAS A) of TcAChE, whereas in hAChE, the benzimidazole group forms π–π interactions with W286 and Y341, as well as in the PAS A site.

In addition, Yoon. et al. synthesized 34 benzimidazole derivatives [63]. Three of the compounds showed good AChE inhibition, the best being compound **5IIc** (Figure 4f), which had an IC_50_ value of 5.12 μM. Furthermore, they made a substitution in the 5-position of the benzimidazole but with a carboxylate, which was not explored by Srikaya et al. However, the best AChE inhibitor found by Yoon et al. also had a substituent in the 2-position of benzimidazole, which was a 4-nitrophenyl substituent. Although Ayse et al. mention that substitution in the 5-position of the benzimidazole ring with electron-withdrawing or electron-donating does not influence eeAChE inhibitory activity, a study by Yoon et al. suggests that the combination of electron-withdrawing or electron-donating in the 5- and 2-positions could influence the activity of the compounds. The use of electron-withdrawing in the 5-position was better than electron-donating and the localization of the hydrocarbon chain with a N atom.

Furthermore, Alpan et al. also reported a series of benzimidazoles with an aromatic ring, searching for compounds to act as anti-AChE, anti-BChE, and antioxidants. Compound **4b** (Figure 4g) was the best anti-AChE, with an IC_50_ value of 0.93 ± 0.04 μM during assays performed with Ellman’s method [64]. Employing molecular docking with the PDB ID: 1EVE, they showed that compound **4b** interacts in the AChE active site and the 5-nitro substituted benzimidazole interacts with W84 and Y130, whereas the 4-hydroxyphenyl interacts with Y121, F290, F330, F331, and Y334, in which the positively charged tertiary amine group goes toward the peripheral site. Therefore, our previous results and those of Ayse et al. suggest that substitution with Cl in the 5-position is better than with a nitro group or a carboxylate, as can be observed in the IC_50_ values obtained (Figure 4).

Faraji et al. evaluated 20 benzimidazoles and benzothiazoles linked to a 1,2,3-triazole ring system [65]. The biological AChE activity was evaluated by employing Ellman’s method and using donepezil as a reference compound. Compound **7e** (Figure 4h), which had the best AChE-inhibitory activity, contains a 1-(2-fluorobenzyl)-1,2,3-triazole linked to a benzimidazole group; it achieves 84% AChE inhibition at 100 μM as opposed to donepezil at 100%, similar to what was reported by Cevik et al. [61]. Docking studies showed that the benzimidazole ring interacts in a hydrophobic pocket with Y333 and F329, as has been reported previously. The 1,2,3-triazole ring makes a hydrogen bond with G122. In addition, the link between the benzimidazole with the 1,2,3-triazole ring was 2-methylenethio.

Also, Can et al. reported a series of 15 benzimidazole derivatives with morpholine to create new derivatives as AChE inhibitors (Table 2) [66].

**Table 2:** Chemical structure reported by Can et al. [66].

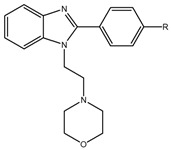		
Compound	R	AChE Inhibition % (103 M)
**2a**	0	21.8
**2b**	0	5.19
**2c**	-Cl	5.48
**2d**	-F	16.2
**2e**	-CH(CH_3_)_2_	17.51
**2f**	-OCH_2_C_6_H_5_	15.75
**2g**	-Br	13.04
**2h**	-N(C_2_H_5_)_2_	10.49
**2i**	-N(CH_3_)_2_	7.54
**2j**	-OC_2_H_5_	7.2
**2k**	-CN	20.45
**2l**	0	33.88
**2m**	0	32.9
**2n**	0	12.21
**2o**	-C_6_H_5_	6.33

**Figure 5:**
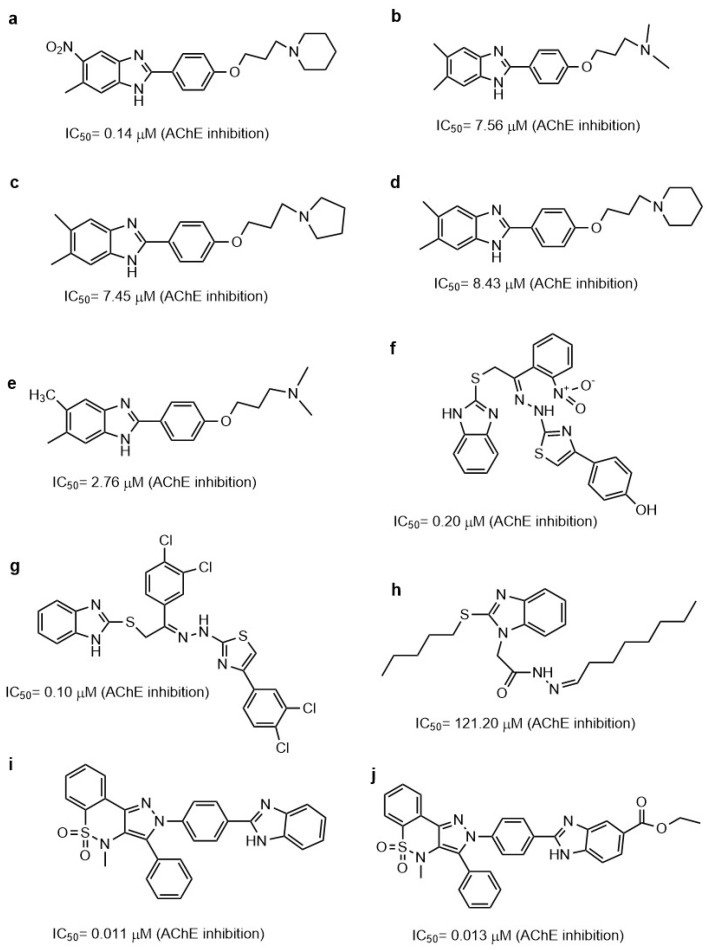
Chemical structures of compounds **A12** (**a**) [67], **A1** (**b**), **A2** (**c**), **A3** (**d**), **A4** (**e**) [68], **16** (**f**), **21** (**g**) [69], **11** (**h**) [70], **12d** (**i**), and **12k** (**j**) [71].

In addition, Sarıkaya et al. reported a series of *o*/*p* propyl phenyl substituted 1H benzimidazoles, with series A having a *p*-substitution and series B having an *o*-substitution [67]. The in silico results by docking employing the PDB ID: 1EVE, AChE showed that the compound for series A had better binding scored in AChE than the compounds from series B. The compound **A12** (Figure 5a) was the best AChE inhibitor; the piperidine ring of this compound interacts with W84 and H440. In addition, the 5-nitro-substituted benzimidazole ring interacts at the PAS, with this compound having an IC_50_ of 0.14 ± 0.021 μM.

A QSAR study recently attempted to find new benzimidazole derivatives to use as AChE inhibitors. El Khatabi et al. conducted a QSAR study and determined that electrostatic and hydrophobic interactions are important for improving AChE-inhibitory activity [68]. Therefore, 36 compounds were designed based on the *o*/*p*-propoxyphenylsubstituted-1H-benzimidazole derivatives developed by Sarikaya et al. The four compounds (**A1**, **A2**, **A3**, and **A4**), designed by taking into consideration the values obtained by QSAR, are shown in Figure 5b–e.

Compound **11** or **A12** (Figure 5a) was the most active; however, all of the designed compounds presented a similar binding energy and binding mode in docking studies (AChE PDB:1EVE). In compound **A1** (Figure 5b), the benzimidazole ring formed a π–π interaction with F331, but the results obtained showed little difference between compounds **11** and **A1**–**A4** (Figure 5b–e). Whether compounds **A1**–**A4** have a tertiary amine from piperidine or a linear tertiary amine from piperazine, their IC_50_ values are very similar.

Furthermore, the benzimidazole ring has been linked to the thiazole ring. Hussian et al. reported 24 compounds as AChE and BChE inhibitors and found that compounds **16** and **21** were the best (Figure 5f,g) [69]. Compound **21**, bearing di-chloro (Cl) groups at the *meta*- and *para*-positions of both phenyl rings **B** and **C**, had IC_50_ values of 0.10 ± 0.05 µM and 0.20 ± 0.05 µM, respectively, whereas compound **16** showed an IC_50_ = 0.20 ± 0.050 µM for AChE. These compounds have better IC_50_ values than the control molecule, donepezil (IC_50_ = 2.16 ± 0.12 µM). The interactions of compound **21** with AChE (PDB:1ACL) were via aromatic residues such as F330, F331, Y334, D72, W84, Y121, and W279. The two Cl atoms on the benzene ring produce a partial positive charge over the benzene ring due to the Cl on both ends of the compound drawing most of the electronic density from the benzene ring. These electronic effects are responsible for compound **21** being a good AChE inhibitor. Compound **16** interacts with AChE residues W84, E199, Y121, W279, D285, Y334, and D72; however, it has electron-donating and electron-withdrawing groups that balance the electronic effect in the molecule. Therefore, electronic density effects could be important during the interactions of these compounds on AChE.

Benzimidazole 2-thiol has also been employed as a medicinal agent. Latif et al. reported a series of these derivatives as having antiradical, anti-AChE, and anti-BChE properties. The best AChE inhibitor was compound **11** with IC_50_ of 121.2 μM (Figure 5h). Compound **11** interacts with the AChE in docking studies (PDB ID: 4EY6 in complex with (-)-galantamine) at an active site with Y121, Y332, Y336, and G79 by hydrogen bonds; and by π–alkyl interactions with W83 and W28, π–donor hydrogen bond with D71, and π–sulfur interactions with Y332 [70]. Comparing the results obtained by Latif et al. and Hussain et al. showed that the substitution of an aromatic ring in the S atom in the 2-position of benzimidazole allows better AChE inhibition than substitution with hydrocarbon chains, even though the molecules reported by Hussain have a higher molecular weight.

Benzimidazole derivatives of a larger size have also been designed and evaluated as AChE inhibitors. Aslam et al. reported a hybrid of pyrazothiazines with benzimidazoles, finding that these compounds have IC_50_ values of nM. Compounds **12d** and **12k** (Figure 5i,j) are the better AChE inhibitors with IC_50_ values of 0.011 ± 0.004 and 0.013 ± 0.004 μM, respectively. These compounds are better than neostigmine and donepezil at 22.2 ± 3.2 and 0.032 ± 0.003, respectively, as evaluated in vitro with Ellman’s method [71].

The binding mode of these compounds was also explored by molecular docking, employing AChE PDB ID:4BDT. When compound **12d** interacts with several aromatic residues, such as Y337 and W86, the phenyl substituent in the pyrazole ring interacts with F297, A204, and F123. The phenyl linker between pyrazolobenzothiazine and benzimidazole forms π–π interactions with Y341; finally, the benzimidazole ring forms π–π interactions with W286 located in the gorge of the AChE catalytic site.

**Figure 6:**
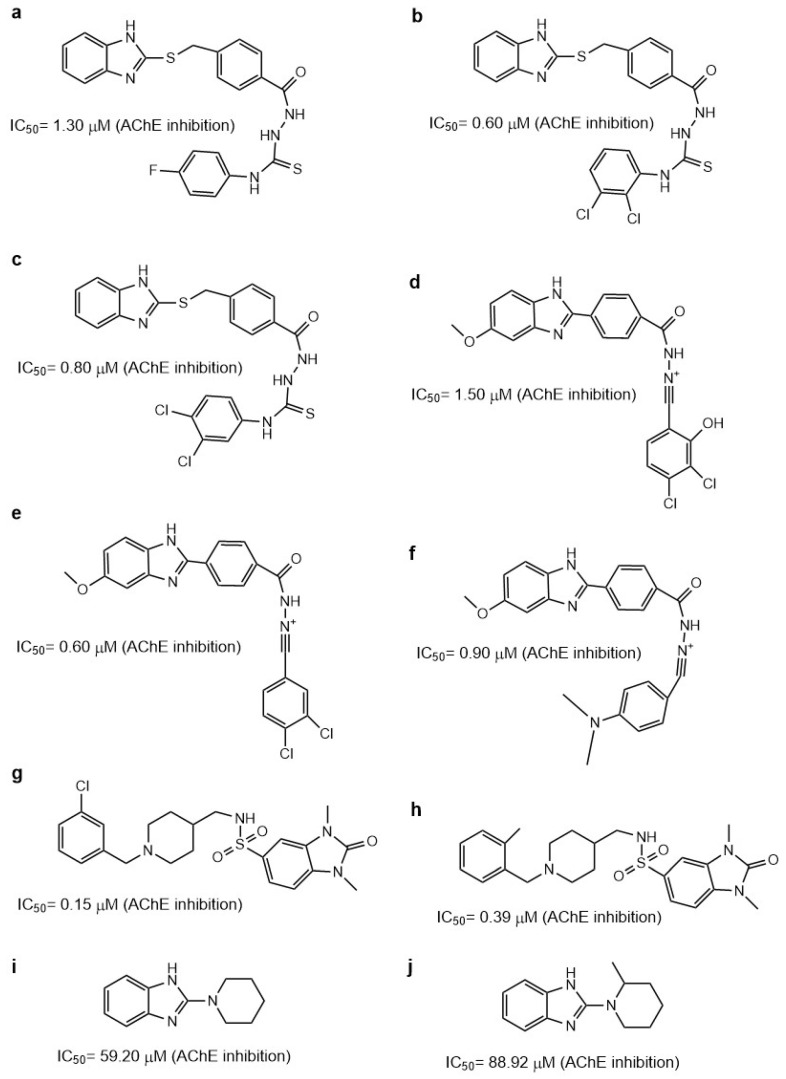
Chemical structures of compounds **1b** (**a**), **1c** (**b**), **1g** (**c**), **2c** (**d**), **2e** (**e**), **2h** (**f**) [72], **15g** (**g**), **15b** (**h**) [73], **12** (**i**), and **13** (**j**) [74].

Adalat et al. reported a series of benzimidazole derivatives with thiosemicarbazide (series 1) and benzimidazole–Schiff bases (series 2). Compounds **1b**, **1c**, **1g**, **2c**, **2e**, and **2h** (Figure 6a–f) had IC_50_ values of 2.4 to 0.60 μM, with the best compounds being anti-AChE **1c** and **2e** [72].

The substitution of series **1** with the aromatic ring with two Cl atoms created better AChE inhibitors, which is in accordance with other works mentioned previously wherein a Cl atom was substituted in the benzimidazole ring in the 5-position. The effect is even better than when a nitro group is used. Similar results were obtained with the derivatives of series **2**, wherein the best compound, **2e**, also has two Cl atoms in the phenyl ring. This compound has the same IC_50_ value as compound **1c** (Figure 6b,e). The authors also mention that the substitution of two Cl atoms at the phenyl ring plays an important role in the AChE inhibition. The molecular docking of compound **1c** shows that the benzimidazole ring forms π–π interactions with W86 and a hydrogen bond with D74, while the dichloro-phenyl ring forms π–π interactions with W286 and Y341, as well as a π–alkyl interaction between Y72 and the Cl atom. These results suggest that this compound is accommodated in the catalytic site of AChE.

Benzimidazolinone derivatives have also been proposed as AChE inhibitors. Mo et al. reported compound **15b** (Figure 6h) as a good AChE inhibitor with an IC_50_ value of 0.39 ± 0.11 μM, employing Ellman’s method [73]. However, when analyzing Table 1 from Mo et al., we see that there are other compounds that have better IC_50_ values as AChE inhibitors, such as **9m** and **15g** (Figure 6g), which have a Cl atom on the aromatic ring in accordance with previous reports. In this work, the authors mention that the activity on AChE was 4Cl (**9m**) > 3F (**9i**) > 3Br (**9o**), showing that the substitution with Cl favored the AChE inhibition. In silico studies using hAChE (PDB ID: 4EY7) showed the benzimidazole ring of compound **15b** interacting with W286 and Y341 in the PAS principally by π–π interactions. Furthermore, the addition of a sulfonyl group to the compounds enhances the AChE-inhibitory activity. This group interacts with F295 and R296 by hydrogen bonds.

Zhu et al. reported 2-aminobenzimidazole as AChE and BChE inhibitors; however, the compounds were more selective for BChE than for AChE. The authors employed Ellman’s method to assay both activities [74]. The crystal structure of AChE employed was PDB ID: 1P0P. The introduction of different hydrocarbon chains to the amino group did not improve the inhibitory activity. However, compound **9** was superior due to having another benzimidazole group and a hydrocarbon chain with an amine group. This compound also has more selectivity for BChE, having an IC_50_ value of 2.26 ± 0.48 μM, and for AChE, with 59.68 ± 0.33 μM. It is important to mention that when a piperidine molecule was introduced to compounds **12** and **13** (Figure 6i,j), they had a better AChE and BChE inhibitory effect, maintaining their BChE selectivity. Their chemical interactions were principally π–π type or via H-bonds.

## 5. Benzazole Compounds as BACE1 Inhibitors

### 5.1. Benzothiazole Derivatives as BACE1 Inhibitors

**Figure 7:**
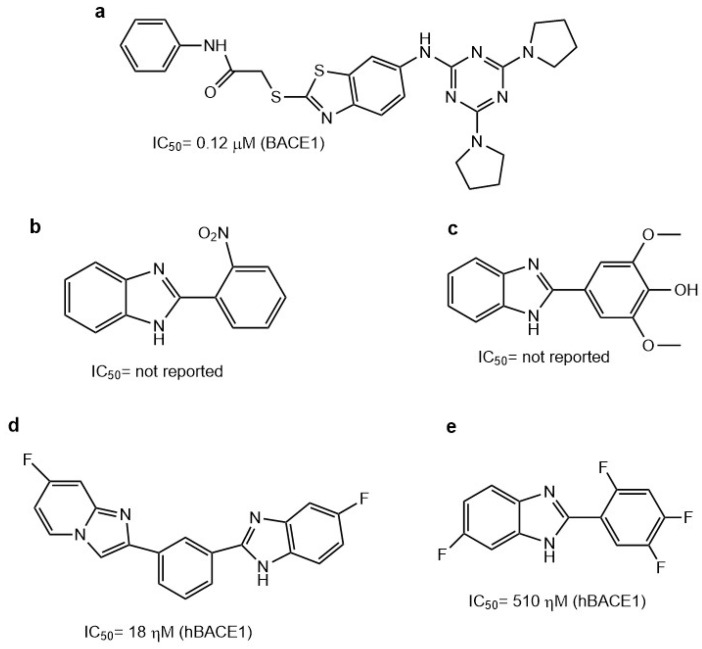
Chemical structures of compounds **5** (**a**) [75], **11** (**b**), **14** (**c**) [76], **34** (**d**) [77], and **7c** (**e**) [78].

Benzothiazoles have been widely employed to detect Aβ aggregation and used as AChE inhibitors; however, there are few reports about their use as BACE1 inhibitors. Wejiun et al. evaluated compounds that contain a benzothiazole group linked to a triazine ring employing a secondary amine group. The best compound was **5**, which contains a pyrrolidinyl in the 4- and 6- positions on the triazine ring and has a phenyl acetamide group in position 4 of benzothiazole (Figure 7a). In molecular docking studies, this compound showed interactions with amino acids (D32, T232, Y198, G230, and Y71) and an IC_50_ value of 0.12 µM [75].

### 5.2. Benzimidazoles as BACE1 Inhibitors

Archana et al. analyzed the interactions of some 2-substituted-1H-benzo(d)imidazole derivatives as BACE1 inhibitors. They found that compounds **11** and **14** were the best BACE1 inhibitors and neuroprotectors (Figure 7b,c). Docking studies that employed PDB ID: 1M4H showed that these compounds interact with the aspartic acid from the catalytic dyads D32 and D228. However, BACE1 inhibition was corroborated neither in vitro nor in vivo [76].

Al-Tel et al. evaluated a series of imidazopyridines with benzimidazole and/or arylimidazole as BACE1 inhibitors [77]. They found that compound **34** (Figure 7d) had an IC_50_ value of 18 nM for BACE1 and was more selective for BACE1 than for BACE2. In addition, the **34**-HCl salt had better solubility in water up to 10 mg/mL. Furthermore, in an in silico study performed employing BACE1 (PDB:2B8L) with compound **34**, it was observed that the compound interacted with the amino acids from the catalytic dyad D32 and D228, as well as with amino acids near the catalytic site, such as T72 and Q328.

Ali et al. evaluated fluoro-benzimidazole derivatives, with compound **7c** (Figure 7e) being the most potent BACE1 inhibitor with a value of IC_50_ = 510 nM measured by a fluorescence resonance energy transfer (FRET) assay [78]. The docking results showed that compound **7c** was binding near to the catalytic dyad of aspartic acids (PDB ID: 1FKN). Furthermore, the authors corroborated that compound **7c** has an effect in vitro and in vivo, diminishing the Aβ plaques on AD mice models; also, the compound is orally bioactive and may allow for brain penetration.

It is important to mention that those compounds that have a chemical structure related to that of benzimidazole have been evaluated as AChE inhibitors. Therefore, it is possible that fluoro-benzimidazole derivatives may act as dual compounds on both targets. In addition, it would be interesting to evaluate all benzazole derivatives that act as AChE inhibitors as BACE1 inhibitors. However, the benzazoles that have been evaluated as a scaffold for BACE1 inhibitors are benzothiazoles and benzimidazoles, but not benzoxazoles.

## 6. Benzazole Compounds Targeting Aβ Aggregation

### 6.1. Benzothiazole Derivatives for Anti-Aβ Aggregation

Benzothiazole molecules have been evaluated as neuroprotective molecules against Aβ toxicity. Cifelli reported three benzothiazole derivatives, which were evaluated in SH-SY5Y neuroblastoma cells for their ability to combat Aβ-induced cell damage. They observed that all the compounds protected against the toxicity and oxidative stress produced by Aβ_1–42_. Therefore, the authors proposed that these compounds could be useful in treating the cellular damage produced in AD and other neurological alterations [79].

Pradhan et al. synthesized a compound binding one benzothiazole moiety with rhodamine (**Rh-BT**). Table 3 shows the chemical structure of this compound; the interactions that occur with Aβ are D7, S8, H6, T10, E11, H13, H14, and Q15. The in vitro assays showed that the compound has low toxicity in PC12 cells and prevents Aβ aggregation. Furthermore, it was observed that **Rh-BT** was able to cross the BBB, having a ΔG of −5.9 kcal/mol [80].

In addition, as mentioned previously, the benzothiazole molecule plays an important role in the identification of amyloid fibrils due to its great affinity to probes such as thioflavin T (Table 3). Therefore, several molecules from benzothiazole have been designed and evaluated for use as optical imaging agents to identify one type of amyloid fibril. Murugan et al. synthesized the compound **BTA-3** (Table 3) and evaluated it as an optical probe, then evaluated its binding to the compound in an Aβ fibril (PDB: 5OQV [81]) and determined its relative binding free energy (ΔG) in different sites. They found four binding sites for **BTA-3** in a beta proto fibril with ΔG values of −46.5, −24.4, −24.8, and −21.3 kcal/mol, having better ΔG binding at site 1 (−46.5). However, the results showed that the molecular and electronic structure of **BTA-3** influences binding sites in the amyloid fibrils [82].

### 6.2. Benzimidazoles for Anti-Aβ Aggregation

Benzimidazoles have also been evaluated for their anti-Aβ aggregation properties but are complexed with iridium (III), ruthenium (II), and platinum (II) (Table 3). The new complexes with iridium avoid the toxicity produced by Aβ_1–42_ on cortical neurons. However, all complexes with the three metals avoid Aβ_1–42_ aggregation [83]. Furthermore, benzimidazole compounds coordinated with Pt (II) and Pd (II) have been shown to interact with Aβ_21–40_, and it is possible that this interaction avoids the cytotoxicity produced by Aβ_21–40_. Some amino acids from the peptide can interact directly with the metal (Pt and Pd) and generate adducts identified by ESI-MS. However, benzimidazole with Au (III) cannot form a direct interaction with the peptide amino acids [84].

Several benzimidazole derivatives have been found to be useful as amyloid imaging probes because of their high binding affinity to Aβ and high uptake into the brain [85].

## 7. Benzazoles as Multitarget Drugs for AD

Benzazoles are molecules with width activities evaluated in different diseases such as antiviral, antibacterial, antimicrobial, fungicidal, antiallergic, antidiabetic, antitumor, anti-inflammatory, anthelmintic, anticonvulsants, antioxidants, antitubercular, antimalarial, as antagonists of the peroxisome proliferator-activated receptor (PPARα), antidepressants, analgesics, central nervous system (CNS) depressants, antileishmanial, antihistamine, between others [86], which implies that benzazoles such as benzothiazoles, benzimidazoles, and benzoxazoles can act on several targets such as benzoxazoles on topoisomerases, kinases, cyclooxygenases, histone deacetylase in cancer, etc. [87], benzothiazoles on tubulin polymerase inhibitor, DNA topoisomerase-inhibitor, an Abl kinase inhibitor, histone deacetylase, inhibitor, Aurora-B kinase inhibitor, etc. [86]. However, some side effects have been associated with benzothiazole exposure such as liver injury [88]. In addition, benzoxazole derivatives are also employed to protect against damage caused by sunlight most absorbing UVA or UVB radiations. Interesting work has been done evaluating the substitution of 2-(phenyl)benzoxazole by an amino group in the 4′ and 5′ positions, showing that the substitution at the 4′ position of the phenyl ring appears to have greater toxicological risks than substituents at the 5′ position. Therefore, it is desirable that during the design and evaluation of benzazole derivatives the toxicological evaluation be considered.

**Figure 8:**
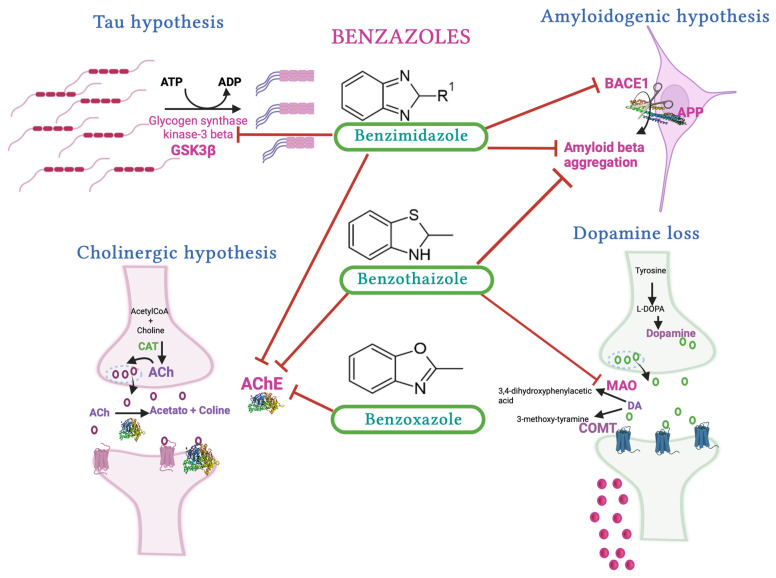
Principal targets to be inhibited by benzazoles as possible multitarget drugs for the treatment of AD. Each row indicated the enzyme or peptide that it inhibited for each benzazole. R^1^ indicated the substitution in the benzazole ring in the 2 position. Figure created with BioRender.com.

As observed before, benzothiazole and benzimidazole are the benzazoles most evaluated in relation to AD, principally as AChE, BChE, BACE1, MAO-A, and MAO-B inhibitors, as well as for their antiradical properties (Figure 8). Benzothiazole derivatives have been shown to be useful as amyloid imaging probes because of their high binding affinity to Aβ aggregates and high uptake into the brain [89]. Benzimidazole has a chemical structure related to that of the indanone group of donepezil, which is an AChE inhibitor used in AD treatment. Furthermore, the binding of a metal to the benzimidazole molecule inhibits Aβ oligomerization. Therefore, benzimidazoles are some of the benzazoles most often evaluated as AChE inhibitors. Also, Gulcan et al. described in a review how some of these compounds are used for different targets involved in AD [90].

Several molecules have been designed to target Aβ_1–42_ production and its oligomerization, some of them principally to mark Aβ, such as Congo red, thioflavin S (a fluorescent probe), and AZD2184 (a PET tracer) (Table 3). Many of these molecules are not able to cross the blood–brain barrier (BBB), despite having a good affinity to Aβ. Therefore, their structures have been imitated in the design of a possible molecule that can cross the BBB. The strategy employed by Ali et al. could be a good model in that they added fluorine atoms to benzimidazole, which improved the ability to cross the BBB [91].

However, we now know which are the principal chemical characteristics in a molecule that help prevent Aβ aggregation. It has been reported that the presence of a tertiary amine in a ring of five carbons helps to establish electrostatic interactions with E22 and D23 of Aβ_1–42_. Moreover, the presence of an aromatic ring helps to establish π–π interactions with F19 and F20 [92]. In addition, K16 is an important amino acid residue that is also able to form hydrophobic interactions between aliphatic substituents in the aromatic rings; its methylene side chain also forms a π–cation interaction with the aromatic ring and its NH_3_ group. As has been observed above for AChE inhibition, it is interesting that these compounds share some chemical characteristics that are also necessary to inhibit βA_1–42_. The use of some benzazoles reported to inhibit AChE or BACE1 could have an anti-Aβ aggregation effect, as could piperidine or pyrrolidinyl rings in the molecules.

**Figure 9:**
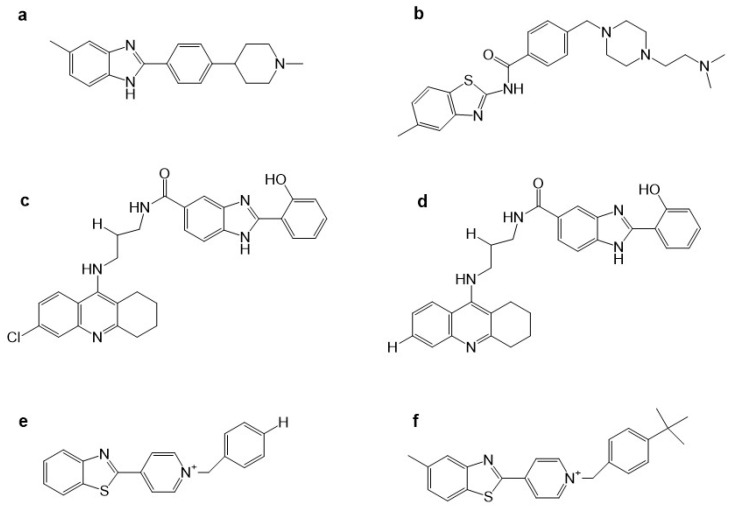
Compound **4f** (**a**) [93], compound **3d** (**b**) [95], compound **TAC-BIM1** (**c**), compound **TAC-BIM2** (**d**) [96], compound **4c** (**e**) and compound **4g** (**f**) [97].

Both benzimidazoles and benzothiazoles have been proposed as multitarget compounds because they can act on AD in different ways, such as via the cholinergic, amyloidogenic, tau, and dopamine pathways. Recently, Karaca et al. synthesized a series of benzothiazole derivatives as multitarget compounds for AD. They have been reported to have dual inhibitory activity on AChE and MAO-A and B. Fourteen benzothiazole derivatives were reported; from these, compound **4f** (Figure 9a) has inhibitory activity against AChE and MAO-B enzymes with IC_50_ values of 23.4 ± 1.1 nM and 40.3 ± 1.7 nM, respectively. In addition, the ability of this compound to inhibit Aβ aggregation was evaluated [93]. Furthermore, compounds derived from benzothiazole-isothioureas were evaluated as multiarget treatment for AD, and it was found that compounds **3f**, **3r**, and **3t** can inhibit AChE activity and Aβ aggregation. The best compound selected was **3t** [94].

Benzimidazole derivatives have also been evaluated as multitarget compounds for AD. Latif et al. synthesized a series of benzimidazole-2-thiol (BT, 2-MBI), which were evaluated for antiradical activity via ABTS and DPPH, and as anti-Alzheimer’s treatment through the evaluation of their AChE and BACE inhibition. Compounds **11** and **14** were able to inhibit AChE and BChE, as well as having antiradical activity [70].

In addition, 2-(4-substituted phenyl)-1H benzimidazoles compounds were synthesized by Unsal-Tan et al., who evaluated these compounds for multitarget treatment against AChE, BChE, and Aβ. Compounds **3d** and **3g**–**i** were able to inhibit BChE but did not reach the catalytic site of AChE, as demonstrated by docking studies. However, compound **3d** (Figure 9b) can be a multitarget compound due to it having inhibitory activity on BChE, preventing Aβ aggregation, and presenting neuroprotection in cells treated with hydrogen peroxide or Aβ_1–40_ [95].

Hybrid molecules from 2-hydroxyphenyl-benzimidazole (BIM) with donepezil and tacrine were also synthesized and evaluated as multitarget compounds for AD. Better activity was observed with hybrid compounds obtained from tacrine (TAC-BIM1 and TAC-BIM2; Figure 9c,d) than for those obtained from donepezil. Different from other works, we evaluated the copper- and zinc-chelating capacity of the compounds and found that the imidazole nitrogen N3 from benzimidazole is involved in this activity. This effect is very important due to this metal participating in the oxidative stress produced by Aβ [96].

Salehi et al. reported on benzylpyridinium-based benzoheterocycles employing benzimidazole, benzoxazole, and benzothiazole to synthesize a compound with inhibitory activity against AChE and BChE, as well as Aβ aggregation. They identified that **4c** and **4g** (Figure 9e and Figure 9f, respectively) could be good multitarget compounds; interestingly, each one has a benzothiazole molecule, showing that this scaffold could be a promising core in a good multitarget compound for AD. In addition, they showed through docking studies that n-benzyl interacts with F329 and D71 and with W83 and H439 by π–π interactions (AChE PDB 1EVE) [97].

Therefore, this work established a starting point for the future rational design based on the structure–multiactivity relationship concept (SMARts) for benzazole compounds to treat AD. For all molecules cited in this review, it is possible to identify the structure that has activity on multiple targets and then establish its multiactivity based on its structure. As shown in Figure 8, we found that the benzimidazole derivatives have activity on four targets, namely GSK3β, BACE1, AChE, and Aβ anti-aggregation, which are targets that have been identified when multitarget compounds for AD have been proposed [98,99]. Then, an in silico study could be done to identify which of these benzimidazole derivatives has better affinity on all these targets identified by examining the participating pharmacophore to establish a SMARts between benzimidazole derivatives and the targets [100].

## 8. Conclusions

Benzazoles can be an important scaffold for multitarget compounds targeting AD. Benzothiazoles and benzimidazoles showed an affinity for AChE π–π interactions with aromatic residues such as F338, W279, and W84, and W286, F295, Y124, Y286, and Y341, respectively. However, compounds with benzimidazoles and triazine molecules have an IC_50_ value on AChE in the nM range, while benzothiazoles have one in the μM range.

Furthermore, compounds with benzothiazoles and benzimidazoles have an affinity for Aβ. In addition, benzimidazoles could be good BACE1 inhibitors due to some molecules showing an IC_50_ value in the nM range. However, there is no information about the use of benzoxazoles as BACE1 inhibitors. Therefore, the evaluation of more benzazoles as BACE1 inhibitors represents an interesting alternative.

Also, benzazoles can act as inhibitors of other proteins, such as MAO-A and MAO-B, or on GSK-3β, in which case they could be used as multitarget drugs.

Therefore, the search for benzazoles that could act as a multitarget treatment for AD may be a good option due to them having some pharmacophores that could inhibit BACE1. Furthermore, a fluor atom being added to these molecules may help them to cross the BBB. Benzimidazole derivatives containing metal atoms could also favor interaction with the catalytic sites of the enzymes and Aβ.

## Figures and Tables

**Table 3:** Benzothiazole and benzimidazole compounds targeting Aβ oligomerization.

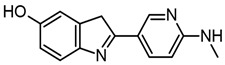 **AZD2184**	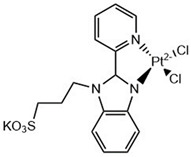 **Pt Compound**
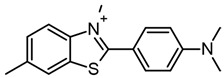 **Thioflavin T**	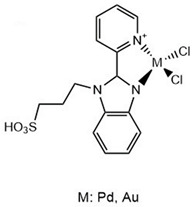 **Pd, Au Compounds**
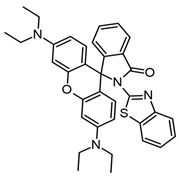 **Compound Rh-BT**	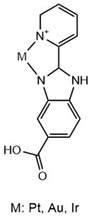 **Pt, Au, Ir Compounds**
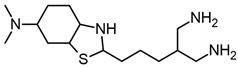 **Compound BTA-3**	

## Data Availability

Not applicable.
